# Quantitative Ultrasound Biomicroscopy Assessment of Anterior Chamber Angle Changes One Year After Laser Peripheral Iridotomy in Primary Angle Closure Suspects

**DOI:** 10.3390/jcm15072544

**Published:** 2026-03-26

**Authors:** Juliette Buffault, Paul Bastelica, Erwan Benouaghrem, Nassima Benhatchi, Christophe Baudouin, Antoine Labbé

**Affiliations:** 1 University Institute of Glaucoma, Quinze-Vingts National Ophthalmology Hospital, IHU FOReSight, 75012 Paris, France; 2 Department of Ophthalmology, AmbroiseParé Hospital, AP-HP, Université de Versailles Saint-Quentin-en-Yvelines, Université Paris-Saclay, 92100 Boulogne-Billancour, France; 3 Clinical Investigation Center (CIC 1423), Quinze-Vingts National Ophthalmology Hospital, IHU FOReSight, INSERM–DGOS, 75012 Paris, France; 4 Department of Ophthalmology, Institute of Glaucoma, Paris-Saint-Joseph Hospital Group, 75014 Paris, France

**Keywords:** anterior chamber, glaucoma, angle closure, gonioscopy, iridotomy, plateau iris, trabecular meshwork, ultrasonography

## Abstract

**Background:** Laser peripheral iridotomy (LPI) is the first-line treatment for eyes with primary angle closure suspect (PACS), but the extent and durability of anterior chamber angle widening over time remain variable. Ultrasound biomicroscopy (UBM) allows detailed quantitative assessment of angle anatomy and underlying mechanisms of residual angle closure. **Methods:** In this prospective cohort study, 39 eyes of 20 PACS patients underwent UBM examination before LPI and at 1 month and 1 year post-procedure. Angle opening distance at 500 µm from the scleral spur (AOD500) and trabecular–ciliary process distance (TCPD) were measured in four quadrants under standardized light conditions. Paired comparisons and linear regression analyses were performed. **Results:** Mean AOD500 increased significantly from baseline (90.1 ± 52.5 µm) to 1 month (146.2 ± 58.2 µm, *p* < 0.001) and remained greater at 1 year (128.4 ± 48.8 µm, *p* < 0.001), with the largest changes observed in the superior quadrant. TCPD remained unchanged over time. Despite a patent iridotomy, iridotrabecular contact (ITC) persisted in 12.8% of eyes at 1 year. Plateau iris configuration was identified in 35.9% of eyes. Eyes with smaller baseline AOD500 showed a more limited anatomical response to LPI, although interaction testing did not reach statistical significance. **Conclusions:** LPI induces significant but partially attenuated anterior chamber angle widening at one year in PACS eyes. A substantial subset exhibits persistent angle closure, frequently associated with plateau iris configuration, underscoring the need for continued post-LPI anatomical surveillance and mechanism-based management.

## 1. Introduction

Primary angle-closure glaucoma (PACG) is a leading cause of irreversible bilateral blindness. Of the nearly 80 million patients with glaucoma worldwide, it has been estimated that one quarter are affected by angle-closure glaucoma [1,2]. The prevalence of PACG and primary angle closure (PAC) is significantly greater in Asian ethnic groups [2,3]. Risk factors for developing PAC include race (Inuit, East Asians), short axial lengths [4], hyperopia, age, gender (2 to 4 times more common in women), and family history.

Angle closure occurs when the peripheral iris apposes the trabecular meshwork, thereby impeding aqueous humor drainage through the anterior chamber angle. The most frequent underlying mechanism is pupillary block, in which resistance at the pupillary margin creates a pressure differential between the posterior and anterior chambers, driving the peripheral iris anteriorly against the trabecular meshwork.

According to the guidelines of the European Glaucoma Society (EGS) [5], primary angle closure suspect (PACS) is defined as two or more quadrants of iridotrabecular contact (ITC), with various possible causes. The term glaucoma (PACG) is used only when both disc and field damage are present with PAC. In a population-based study [6], 22% of PACS patients progressed to PAC, and 28.5% of PAC patients developed PACG within 5 years if no treatment was given.

Laser peripheral iridotomy (LPI) is the first-line treatment for PACG and is also performed prophylactically in eyes at risk of acute angle-closure. The neodymium-doped yttrium aluminium garnet (Nd:YAG) laser creates a full-thickness opening in the peripheral iris, establishing direct communication between the posterior and anterior chambers, relieving pupillary block, reducing iris convexity, and widening the anterior chamber angle.

A randomized controlled trial demonstrated that prophylactic LPI significantly reduced the incidence of angle-closure events in primary angle-closure suspects, although the absolute risk reduction was modest, underscoring the heterogeneity of anatomical responses to iridotomy [7].

In some cases, iridotomy is insufficient to open the angle. This is the case when angle closure occurs without pupillary block. Iridotrabecular apposition or synechiae can result from the iris and/or lens being pushed, rotated, or pulled forward for a variety of reasons. In iris plateau configuration, angle closure is induced by anteriorly positioned ciliary processes which push the peripheral iris forwards [8]. An evaluation of the anterior chamber angle anatomy behind the closure mechanism is therefore essential since it will define the most appropriate treatment methods. It is sometimes necessary, however, to perform the iridectomy as much for diagnostic purposes as for therapeutic ones. For example, the diagnosis of plateau iris can be confirmed only when a patent iridectomy fails to change peripheral iris configuration and relieve angle closure [9].

Ultrasound biomicroscopy (UBM) enables high-resolution, quantitative assessment of anterior segment structures inaccessible to clinical examination, including the ciliary body and retro-iris space, making it particularly valuable for characterizing angle-closure mechanisms and monitoring post-LPI anatomical changes [10,11,12]. UBM has also been established as a qualitative tool for evaluating glaucoma-related anterior segment pathology [13]. Recent longitudinal imaging studies have shown that anatomical widening of the anterior chamber angle after LPI may attenuate over time, emphasizing the importance of evaluating the durability of post-LPI anatomical changes [14]. Furthermore, UBM-based assessment has been shown to improve the predictability of LPI efficacy in primary angle closure [15].

The aim of this study is to evaluate iridocorneal angle changes one year after LPI for narrow angles, using UBM, and then to define the characteristics of eyes in which appositional angle closure persists after LPI.

## 2. Materials and Methods

### 2.1. Patients

Thirty-nine eyes of 20 consecutive patients diagnosed with PACS were evaluated by UBM before and after LPI. The study was conducted from September 2009 to January 2011 at Ambroise Paré Hospital in Boulogne-Billancourt, France. The study was conducted in accordance with the principles of the Declaration of Helsinki. All patients were informed of the nature and purpose of the examinations and procedures and provided informed consent prior to inclusion. The subjects were diagnosed PACS after gonioscopy (appositional contact between the peripheral iris and trabecular meshwork, trabecular meshwork not visible on gonioscopy, intraocular pressure in the normal range ≤ 21 mmHg, no peripheral anterior synechiae [PAS]) and UBM. They did not present any signs of PACG. Patients presenting any pathology of secondary angle closure, such as uveitis, iris neovascularization, traumatism, tumor, or intumescent lens, were excluded. All the patients selected underwent LPI performed with a Nd:YAG laser.

### 2.2. Gonioscopy

Gonioscopy was performed before LPI by an experienced examiner using a Goldmann mirror lens, and anterior chamber angles were graded in all four quadrants according to the Shaffer classification [16]. Post-LPI gonioscopic evaluation was performed at 1 month and 1 year to verify iridotomy patency and assess residual angle narrowing or the development of PAS.

### 2.3. Ultrasound Biomicroscopy

UBM was performed using a 50 MHz transducer (Quantel-Médical, Aviso^®^, Cournon-d’Auvergne, France). The exploration window measured 5 mm × 10 mm, yielding images with a 50 µm resolution. All UBM measurements were performed by a single experienced examiner (N.B.) with the patient in a supine position in dark and light room conditions, eyes open, after application of a drop of oxybuprocaine and a gel to the eye to create an interface and improve image resolution. Variation in accommodation was minimized by asking the patient to fixate the fellow eye on a specific target on the ceiling. Radial images of the superior, inferior, nasal, and temporal quadrants and one image centered on the anterior chamber were acquired.

Standardized acquisition conditions were maintained throughout the study. Although formal intraobserver repeatability testing was not performed, published data on UBM measurement reproducibility support the reliability of a single-examiner protocol for AOD and TCPD measurements [17].

**Figure 1:**
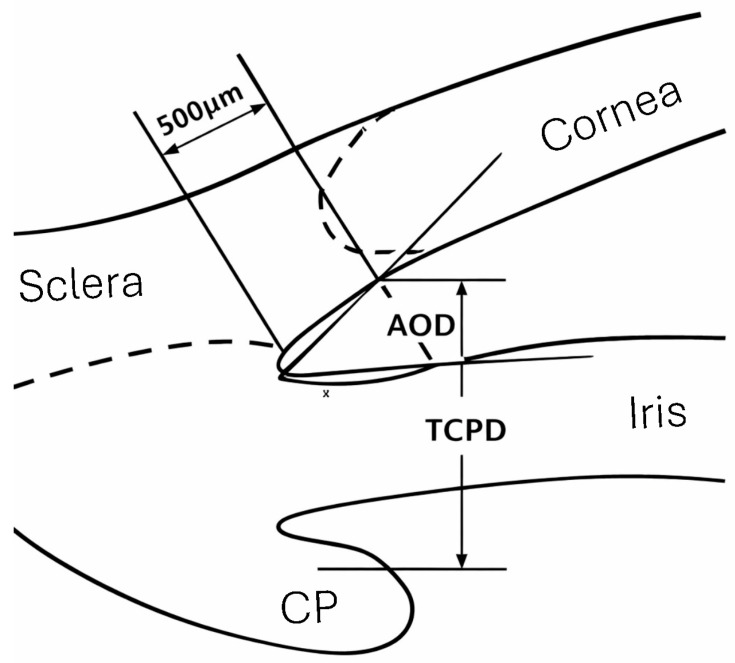
Ultrasound biomicroscopy scheme of a quadrant of the eye. AOD500 was measured on the line perpendicular to the trabecular meshwork at a point 500 µm from the scleral spur. TCPD was measured on the line extending from a point 500 µm from the scleral spur perpendicularly through the iris to the ciliary process (CP).

The angle opening distance at 500 µm from the scleral spur (AOD500) and trabecular–ciliary process distance (TCPD) were measured in the superior, inferior, nasal, and temporal quadrants before LPI and after LPI at 1 month and 1 year, in light conditions (Figure 1). AOD500 is the distance from the corneal endothelium to the anterior iris surface, measured perpendicular to a line drawn along the trabecular meshwork, 500 µm from the scleral spur. TCPD was measured on a line extending from the corneal endothelium 500 µm from the scleral spur, perpendicular from the iris to the ciliary process. Plateau iris was defined if all the following criteria [18] were fulfilled in at least 2 quadrants:

1. The ciliary process was anteriorly oriented, supporting the peripheral iris.
2. The iris root had a steep rise from its point of insertion, followed by a downward angulation from the corneoscleral wall.
3. Presence of a central flat iris plane.
4. An absent ciliary sulcus.
5. Irido-angle contact (above the level of the scleral spur) in the same quadrant.

### 2.4. Laser Iridotomy

Pilocarpine 2% was instilled 30 min before the procedure to constrict the pupil. Laser iridotomy was performed with an Nd:YAG laser (YC-1800; NIDEK, Gamagori, Japan) by a single operator on all patients. Oxybuprocaine 0.5% and apraclonidine 1% were dropped into the eye prior to surgery, and then an iridotomy lens was inserted into the eye to excise the superotemporal or superonasal area of the iris. Nd:YAG laser irradiation was applied 3–10 times at 2.3–3.4 mJ. After surgery, apraclonidine 1% was dropped into the eye and then indomethacin was administered three times a day for a week. Post-LPI evaluations were carried out 1 month and 1 year after LPI.

### 2.5. Data Analysis

Statistical analysis was performed using GraphPad Prism (GraphPad Software version 10.2.2, San Diego, CA, USA). Paired statistical tests were used to compare pre-LPI measurements with post-LPI measurements at 1 month and 1 year. Continuous variables are reported as mean ± standard deviation (SD). An interaction term between baseline AOD500 category and time after LPI was included in linear regression models to assess differential anatomical response. All statistical tests were two-sided, and a *p*-value < 0.05 was considered statistically significant.

Given that some patients contributed measurements from both eyes, eye-level analysis was retained as the primary approach for quantitative UBM parameters (AOD500 and TCPD), consistent with prior UBM studies in angle-closure disease [19,20]. However, for features with an inherently bilateral anatomical basis—namely plateau iris configuration and persistence of ITC—results were additionally reported at the patient level (i.e., proportion of patients with at least one affected eye), as these conditions likely reflect individual anatomical predispositions rather than independent eye-level events. It should be noted that measurements from fellow eyes of the same patient are not fully independent, and the absence of a mixed-effects model or generalized estimating equations (GEE) approach to formally account for inter-eye correlation in the quantitative analyses is acknowledged as a limitation of the present study.

## 3. Results

Thirty-nine eyes of 20 PACS patients were enrolled in this study. There were 13 women and 7 men, with a mean age of 57.2 ± 13.9 years. No serious adverse events were observed after LPI; mild adverse events were limited to transient postoperative inflammation (N = 3 eyes).

Before LPI, gonioscopic examination confirmed narrow anterior chamber angles in all eyes. Mean Shaffer grades were lowest in the superior quadrant (0.75 ± 1.14), followed by the inferior (1.33 ± 0.98), temporal (1.42 ± 1.00), and nasal quadrants (1.58 ± 0.79). These findings indicate a preferential superior angle narrowing, consistent with UBM measurements (Table 1).

Plateau iris configuration was diagnosed in 14 of 39 PACS eyes (35.9%). When analyzed at the patient level, 11 of 20 patients (55%) had plateau iris configuration affecting at least one eye. Three eyes (7.7%) still exhibited ITC in at least two quadrants at 1 month after LPI, and five eyes (12.8%) showed persistent ITC in two or more quadrants at 1 year. At the patient level, this corresponded to 2 of 20 patients (10%) at 1 month and 3 of 20 patients (15%) at 1 year.

**Figure 2:**
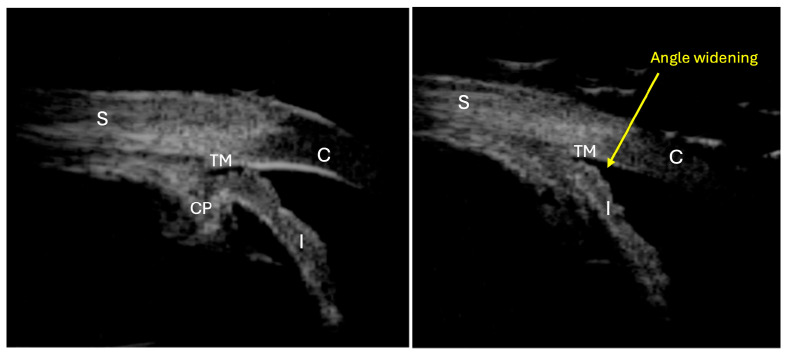
UBM photographs of the inferior angle before (**left**) and after (**right**) LPI. Anatomical structures are labeled: sclera (S), cornea (C), iris (I), trabecular meshwork (TM), and ciliary process (CP). The yellow arrow on the post-LPI image indicates the site of angle widening. The images show flattening of the iris and opening of the angle.

UBM analysis showed that AOD500 increased most prominently in the superior quadrant at 1 month (45.6 ± 56.9 to 118.5 ± 70.0 µm; *p* = 0.002) and remained significantly larger at 1 year (73.0 ± 49.9 µm; *p* = 0.002). Significant increases were also observed in the inferior (*p* = 0.016 at 1 month; *p* < 0.001 at 1 year) and nasal quadrants (*p* < 0.001 at 1 month; *p* = 0.033 at 1 year). The temporal quadrant showed a numerical increase but did not reach statistical significance at 1 month (*p* = 0.073) or 1 year (*p* = 0.096). Across quadrants, mean AOD500 increased from 90.1 ± 52.5 µm at baseline to 146.2 ± 58.2 µm at 1 month after LPI (*p* < 0.001) and remained increased at 128.4 ± 48.8 µm at 1 year (*p* < 0.001) (Figure 2, Table 1).

The TCPD remained unchanged after LPI, with mean values of 0.67 ± 0.30 mm at baseline, 0.69 ± 0.35 mm at 1 month, and 0.68 ± 0.30 mm at 1 year, with no statistically significant differences over time.

**Figure 3:**
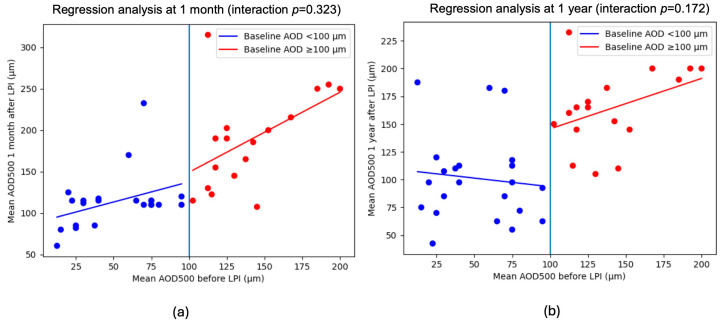
Mean AOD500 before LPI and (**a**) 1 month after LPI and (**b**) 1 year after LPI.

Although statistically significant, angle widening in eyes with baseline AOD500 ≤ 100 µm was modest and markedly lower than in eyes with wider baseline angles. Linear regression analysis demonstrated a different relationship between baseline and post-LPI mean AOD500 according to baseline angle width. Eyes with baseline mean AOD500 < 100 µm showed a shallow regression slope, indicating minimal anatomical response to LPI, whereas eyes with baseline mean AOD500 ≥ 100 µm exhibited a steeper positive relationship. In an interaction model comparing the two regression slopes, the difference did not reach statistical significance at 1 month (*p* = 0.32) or at 1 year (*p* = 0.17), although a consistent trend toward a stronger response in eyes with wider baseline angles was observed (Figure 3).

## 4. Discussion

In this cohort of predominantly Caucasian eyes with PACS and normal baseline IOP, Nd:YAG LPI produced a substantial anatomical opening of the anterior chamber angle that was maximal at 1 month and partially regressed by 1 year, while remaining significantly wider than baseline.

These findings are consistent with prior UBM studies showing that LPI promptly relieves the pupillary block component of angle closure, with the greatest effect shortly after the procedure [19,20,21,22]. The partial loss of angle widening over time observed in our data aligns with the concept that, beyond pupillary block, other anatomic drivers (lens-related crowding and anteriorly positioned ciliary processes) may continue to contribute to long-term angle configuration.

An important consideration is the distinction between primary angle-closure suspects and more advanced stages of angle-closure disease. In PACS, ITC is typically intermittent and reversible and the trabecular meshwork is presumed to be structurally and functionally preserved. In contrast, in PAC or PACG, repeated or prolonged appositional closure and the development of PAS may result in chronic trabecular dysfunction [5]. As a consequence, the anatomical and functional response to LPI may differ substantially across disease stages. While LPI effectively relieves pupillary block in PACS eyes, its impact on angle configuration and aqueous outflow may be more limited in eyes with established trabecular damage. The present study specifically focused on PACS eyes, but the results should not be extrapolated to more advanced forms of angle-closure disease.

Recent critical appraisals have questioned the universal use of prophylactic LPI in primary angle-closure suspects, highlighting the relatively low absolute risk of progression and the need for improved anatomical risk stratification [23].

A clinically important observation is that a meaningful proportion of eyes continued to show appositional ITC despite a patent iridotomy (12.8% of eyes at 1 year), corresponding to 15% of patients with persistent contact in at least one eye. Persistence of contact after LPI has been repeatedly reported, particularly in populations with anatomically narrow angles [20,24]. In the Liwan Eye Study, for example, ITC persisted in a large fraction of eyes after LPI (59% in at least one quadrant and 20% in ≥3 quadrants) and was associated with smaller angle dimensions and thicker iris features, supporting a mechanistic role for non-pupil-block contributors in maintaining closure [20]. Our cohort extends these findings to a European setting and longer follow-up within routine clinical practice, reinforcing that successful iridotomy should not be interpreted as definitive resolution of the angle-closure phenotype.

Our study also identified a high prevalence of plateau iris configuration (35.9%), which provides a plausible anatomical explanation for incomplete angle opening after LPI in a subset of eyes. The proportion we observed is broadly in line with UBM-based reports showing that plateau iris mechanisms are common among angle-closure suspects, with a reported prevalence ranging from approximately 20% to 32% across studies [18,25,26,27,28]. Plateau iris represents a primarily anterior mechanism in which a patent iridotomy may flatten the iris bombé without adequately widening the peripheral angle, leaving residual appositional closure or a narrow angle susceptible to further closure under mydriasis or physiologic pupillary dilation.

Gonioscopy remains the clinical reference standard for assessing the anterior chamber angle and was performed both before and after LPI in all patients. However, gonioscopy has inherent limitations in characterizing the structures posterior to the iris, particularly the ciliary body and the mechanisms of non-pupil-block closure. UBM uniquely provides high-resolution imaging of these posterior segment structures, enabling objective identification of plateau iris configuration and quantitative measurement of angle parameters without contact pressure artifact. The complementary use of gonioscopy and UBM, as implemented in this study, is supported by comparative data showing good agreement between the two modalities for angle-closure grading while highlighting the additional mechanistic information provided by UBM [29].

These data have direct implications for contemporary management, particularly in light of evidence supporting earlier lens-based intervention in angle-closure disease. The EAGLE randomized trial demonstrated that early clear-lens extraction achieved better IOP outcomes, improved patient-reported quality of life, and was more cost-effective than standard care (which included LPI) in patients with PAC and PACG (with elevated IOP and/or glaucomatous damage) [30]. While our cohort differs materially because eyes were selected with normal IOP and without established glaucomatous damage, the EAGLE results underscore a key concept: when lens-related crowding or persistent angle narrowing remains after LPI, lens extraction can address the anatomic crowding mechanism more definitively than iridotomy alone. Therefore, in eyes demonstrating persistent occludable angles after LPI (particularly when accompanied by lens thickening, shallow anterior chamber, or progressive PAS), early consideration of lens extraction may be clinically rational.

For eyes in which residual closure is predominantly attributable to plateau iris configuration, adjunctive peripheral iridoplasty remains a relevant option. Evidence suggests that argon laser peripheral iridoplasty can widen the angle in plateau iris and persistent appositional closure after LPI, although durability and comparative effectiveness vary across studies and the evidence base includes heterogeneous designs [31]. Clinically, our results support a mechanism-based escalation pathway: LPI as first-line for suspected pupillary block, followed by targeted adjunctive therapy (iridoplasty for plateau iris behavior; lens extraction when lens-related crowding dominates), rather than relying on LPI alone as a definitive solution.

Several limitations should be recognized. The study is single-center with a moderate sample size, and although the follow-up captures meaningful anatomical evolution over one year, longer-term outcomes (PAS development, IOP trajectory, and conversion to PACG) were not assessed. UBM metrics, while informative, depend on imaging conditions and scleral spur identification, and we did not incorporate additional biometric predictors such as lens vault or anterior chamber volume that may better stratify lens-driven mechanisms. Measurements from fellow eyes of the same patient are not fully independent, and the absence of a mixed-effects model or GEE approach to formally account for inter-eye correlation in the quantitative analyses is acknowledged as a limitation. Finally, the cohort was restricted to Caucasian eyes, limiting generalizability to populations at higher baseline risk of angle closure.

Future research should address several of the limitations identified in this study. First, longer-term prospective follow-up (3–5 years) is needed to characterize the durability of post-LPI angle widening and to determine whether patients with persistent ITC or plateau iris configuration progress to PAC or PACG at higher rates. Second, the integration of anterior segment optical coherence tomography (AS-OCT) alongside UBM would enable non-contact, three-dimensional assessment of angle parameters across larger patient cohorts [32]. Third, the identification of baseline predictors of insufficient response to LPI—including lens vault, anterior chamber depth, and ciliary body morphology—would allow better risk stratification and individualized treatment decisions. Fourth, prospective head-to-head comparisons between LPI and primary lens extraction in PACS eyes with unfavorable anatomical profiles (high lens vault, shallow anterior chamber) are warranted to guide management.

## 5. Conclusions

LPI produces significant and sustained (though partially attenuated) angle widening at one year in eyes with narrow angles, but a clinically meaningful minority retain appositional closure and a high proportion exhibit plateau iris configuration. Therefore, patients who have undergone laser iridotomy should not be considered cured. Post-LPI surveillance with gonioscopy supplemented by UBM when residual or recurrent narrowing is suspected remains essential to identify persistent non-pupil-block mechanisms.

## Figures and Tables

**Table 1:** Angle Opening Distance (AOD500, µm) using Ultrasound Biomicroscopy before and after Laser Peripheral Iridotomy (LPI).

Quadrant	Before LPI (Mean [SD]; n)	After 1 Month (Mean [SD]; n)	*p* (vs. Before)	After 1 Year (Mean [SD]; n)	*p* (vs. Before)
Superior	45.6 [56.9]; n = 39	118.5 [70.0]; n = 39	0.002	73.0 [49.9]; n = 39	0.002
Inferior	83.6 [62.6]; n = 39	159.2 [96.2]; n = 39	0.016	157.2 [76.6]; n = 39	<0.001
Nasal	114.1 [77.4]; n = 39	176.9 [78.0]; n = 39	<0.001	152.8 [63.3]; n = 39	0.033
Temporal	116.9 [65.6]; n = 39	176.9 [96.1]; n = 39	0.073	142.1 [68.9]; n = 39	0.096
AOD500 (mean)	90.1 ± 52.5; n = 39	146.2 ± 58.2; n = 39	<0.001	128.4 ± 48.8; n = 39	<0.001

## Data Availability

Data are available from the corresponding author upon reasonable request.

## Abbreviations

The following abbreviations are used in this manuscript:

AOD500	Angle Opening Distance at 500 µm from the scleral spur
AS-OCT	Anterior segment optical coherence tomography
EGS	European Glaucoma Society
GEE	Generalized estimating equations
IOP	Intraocular pressure
ITC	Iridotrabecular contact
LPI	Laser peripheral iridotomy
Nd:YAG	Neodymium-doped yttrium aluminium garnet
PAC	Primary angle closure
PACG	Primary angle-closure glaucoma
PACS	Primary angle closure suspect
PAS	Peripheral anterior synechiae
TCPD	Trabecular–ciliary process distance
UBM	Ultrasound biomicroscopy
